# A national perspective on exposure to essential surgical procedures among medical trainees in Nigeria: a cross-sectional survey and recommendations

**DOI:** 10.1186/s12909-023-04847-4

**Published:** 2023-11-30

**Authors:** Paul Tunde KingPriest, Barnabas Tobi Alayande, Emmanuel Walong Clement, Mustapha Muhammed, Joy Ohejem Egbiri, Miracle Shanabo, Etinosa Kevin Osayande, Abiodun Ayomide Atunrase, Jamiu Israel Abubakar, Daniel Chukwuma Eze, Stephen Adekoya, Gideon Bulus Chiroma, Onosegbe Moses Aikhuomogbe, Fatima Shuwa Gaila, Dennis Yaga, Nomsu Noble Thomas, Chukwudi Anthony Chukwunta, Matthew T. Hey, Callum Forbes, Robert R. Riviello, Bashiru O. Ismaila

**Affiliations:** 1 Surgical Equity and Research Hub, Jos, Nigeria; 2 The Global Health Network, Centre for Tropical Medicine and Global Health, University of Oxford, Oxford, UK; 3 Center for Equity in Global Surgery, University of Global Health Equity, Kigali, Rwanda; 4 Harvard TH Chan School of Public Health, Boston, USA; 5 University of Jos, Jos, Nigeria; 6 Usman Danfodio University, Sokoto, Nigeria; 7 Benue State University, Makurdi, Nigeria; 8 Bingham University Teaching Hospital, Jos, Nigeria; 9 University of Benin, Benin, Nigeria; 10 Afe Babalola University, Ado-Ekiti, Nigeria; 11 University of Nigeria, Nsukka, Nigeria; 12 University of Ibadan, Ibadan, Nigeria; 13 Olabisi Onabanjo University, Sagamu, Nigeria; 14 Gombe State University, Gombe, Nigeria; 15 Ambrose Alli University, Ekpoma, Nigeria; 16 University of Maiduguri, Maiduguri, Nigeria; 17 Kaduna State University, Kaduna, Nigeria; 18 Madonna University, Elele, Nigeria; 19 Enugu State University of Technology, Enugu, Nigeria; 20 Program in Global Surgery and Social Change, Harvard Medical School, Boston, USA; 21 Jos University Teaching Hospital, Jos, Nigeria

**Keywords:** Procedures, Nigerian medical graduates, Procedural exposure, Confidence, Geopolitical zones

## Abstract

**Background:**

In sub-Saharan Africa, recent graduates from medical school provide more direct surgical and procedural care to patients than their counterparts from the Global North. Nigeria has no nationally representative data on the procedures performed by trainees before graduation from medical school and their confidence in performing these procedures upon graduation has also not been evaluated.

**Methods:**

We performed an internet-based, cross-sectional survey of recent medical school graduates from 15 accredited Federal, State, and private Nigerian medical schools spanning six geopolitical zones. Essential surgical procedures, bedside interventions and three Bellwether procedures were incorporated into the survey. Self-reported confidence immediately after graduation was calculated and compared using cumulative confidence scores with subgroup analysis of results by type and location of institution. Qualitative analysis of free text recommendations by participants was performed using the constant comparative method in grounded theory.

**Results:**

Four hundred ninety-nine recent graduates from 6 geopolitical zones participated, representing 15 out of a total of 44 medical schools in Nigeria. Male to female ratio was 2:1, and most respondents (59%) graduated from Federal institutions. Students had greatest practical mean exposure to bedside procedures like intravenous access and passing urethral foley catheters and were most confident performing these. Less than 23% had performed over 10 of any of the assessed procedures.

They had least exposures to chest tube insertion (0.24/person), caesarean Sect. (0.12/person), and laparotomy (0.09/person). Recent graduates from Federal institutions had less procedural exposure in urethral catheterization (*p* < 0.001), reduction (*p* = 0.035), and debridement (*p* < 0.035).

Respondents that studied in the underserved North-East and North-West performed the highest median number of procedures prior to graduation. Cumulative confidence scores were low across all graduates (maximum 25/60), but highest in graduates from Northern Nigeria and private institutions. Graduates recommended prioritizing medical students over senior trainees, using simulation-based training and constructive individualized non-toxic feedback from faculty.

**Conclusion:**

Nigerian medical students have poor exposure to procedures and low confidence in performing basic procedures after graduation. More attention should be placed on training for essential surgeries and procedures in medical schools.

**Supplementary Information:**

The online version contains supplementary material available at 10.1186/s12909-023-04847-4.

## Background

**Fig. 1:**
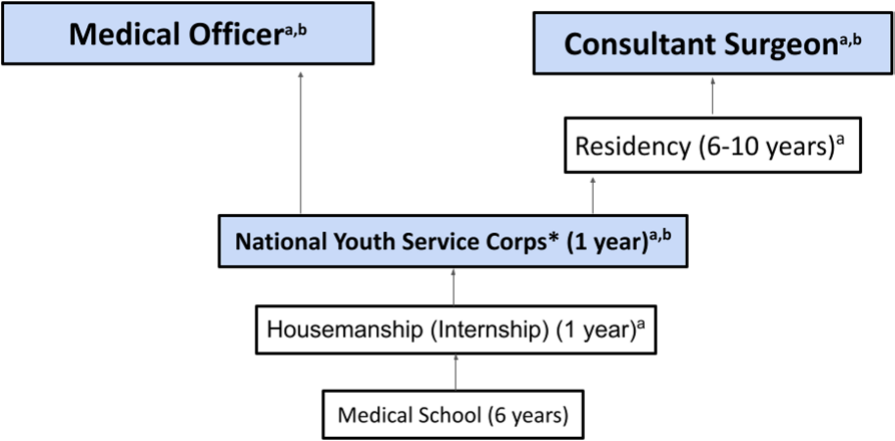
Medical training and the potential for provision of surgery without supervision in Nigeria. **a** Provide operative surgical and other procedural care. **b** Can provide independent procedural and surgical care without direct supervision

Globally, approximately 143 million additional surgeries are needed per year to prevent mortality and disability, and these deficits are more marked in Low- and Middle-Income Countries (LMICs) [1]. The high burden of surgical disease, coupled with an insufficient number of surgical, anaesthesia, and obstetrics specialists in LMICs is a critical contributing factor to this challenge. For instance, Nigeria’s surgical specialist density is 1.8 per 100,000 and significantly falls short of the Lancet Commission’s recommendation of 20 per 100,000 [2]. Inadequate access to surgical care should be considered a global public health crisis, and creative measures should be pursued to address this [3]. In parts of sub-Saharan Africa, recent medical graduates are often thrust to the front lines of clinical care before undergoing specialist training (Fig. 1), unlike their High-Income Country counterparts who mandatorily require residency training [4–7]. In addition to basic bedside procedures like catheterization, intravenous cannulation, and control of haemorrhage with pressure dressings, medical school graduates are often faced with the need to perform basic surgical procedures ranging from suturing of traumatic wounds and chest tube insertion to performing emergency caesarean sections as non-specialist physicians [8]. Undergraduate surgical training in variable resource contexts should prepare non-specialist practitioners to carry out surgical procedures within their scope of practice [7, 9, 10].

In Nigeria, the state of undergraduate medical education has been described [10–12]. After completing medical school, graduates begin their practice by mandatorily working as house officers (interns) at a secondary or tertiary health facility for one year [13, 14]. During this period, interns are temporarily licensed to work under the supervision of consultant physicians across 4 specialities- Surgery, Obstetrics Gynaecology, Internal Medicine and Paediatrics [14]. They are positioned as the first line of patient management in these facilities and carry out basic resuscitation, perioperative patient care, bed-side procedures and selected essential surgical procedures. Efficiency as an intern requires procedural skills which should have been gained during medical school training [14]. Shortly after this, medical school graduates are given a permanent practising licence and are posted across primary, secondary, and tertiary care facilities as a part of the National Youth Service Corps [15]. During this year, many graduates practice independently at primary or secondary facilities without specialist supervision and perform bedside procedures like catheterization and intravenous access, essential obstetric procedures like uterine evacuation, and facility level surgical procedures, including the World Bank proposed list of surgical procedures essential at the first (primary health centre), second (district hospital), and tertiary (teaching hospital) levels of health care [3]. Non-specialist doctors contribute to providing these procedures, most especially at secondary and tertiary care facilities during their year of supervised internship, and at primary and secondary healthcare levels during their national service year.

## Methods

The aim of this study was to identify gaps in exposure of recent graduates from Nigerian medical schools to surgical procedural skills by assessing the self-reported type and number of procedures performed during training. We also aimed to assess their self-perceived level of confidence in their ability to carry out these procedures upon graduation, comparing outcomes from private, Federal, and State-owned medical schools in Nigeria. The psychological concept of self-efficacy is strongly linked with self-perceived confidence [16]. Even though self-reported confidence has not been formally correlated with competence in practice, it has been found to be an essential prerequisite for junior doctors to be able to engage in clinical activities fully [17]. In addition, low self-confidence has been linked with increased burn-out and higher levels of performance anxiety [18]. Uncertainty about how to perform basic procedures is common with recent graduates and the incorporation of confidence building into procedural skills trainings may be even more important for undergraduate medical training [19].

### Study population

There were 37 fully accredited, and 7 partially accredited medical colleges in Nigeria (making a total of 44) as at the time of the study [20]. Accredited schools were graduating a total of 3,530 medical doctors per year into the pool of medical practice. Of these, 6 are private, 14 are public State-owned, and 17 are public, Federal-owned institutions (Table 1) [20]. This study was a descriptive, cross-sectional, observational, online survey of recently graduated medical doctors from accredited medical schools across the six geopolitical zones of Nigeria. National accreditation was defined by inclusion on the Medical and Dental Council of Nigeria (MDCN) accreditation list at the time of study, while recent graduates referred to the last graduated set of medical students from the medical schools (as of December 2021). We defined ‘recent medical graduates’ as the most recent set (academic cohort) of students that had graduated as medical doctors from Nigerian medical schools. We placed a limit of 3 years on this definition and kept the years of graduation within the range of years 2019 to 2021 to mitigate recall bias from longer times from graduation. Incorporating medical doctors with a limit of 2019 into this definition was necessary because some medical schools did not graduate any medical doctors in recent years due to university staff and health worker industrial strike actions.

**Table 1:** Accredited Medical Schools in Nigeria

Geopolitical Zone	Name of Institution	MDCN Quota^a^	Ownership	Teaching Hospital Bed Capacity (August 2022)
**North East (2 medical schools)**	College of Medical Sciences, University of Maiduguri, Borno State	150	Federal	1200
	College of Health Sciences, Gombe State University	60	State	500
**North West (4 medical schools)**	College of Medicine, Ahmadu Bello University Zaria, Kaduna State	180	Federal	
	Faculty of Medicine, Bayero University Kano, Kano State	150	Federal	500
	College of Health Sciences, Usmanu Danfodio University Sokoto, Sokoto State	150	Federal	900
	College of Medicine, Kaduna State University, Kaduna Sate	60	State	500
**North Central (5 medical schools)**	College of Medicine, University of Ilorin, Kwara State	150	Federal	600
	College of Medical Sciences, University of Jos, Plateau State	150	Federal	620
	College of Health Sciences, Bingham University Karu, Nasarawa State	50	Private	250
	College of Health Sciences, Benue State University, Makurdi, Benue Stae	75	State	350
	College of Health Sciences, University of Abuja	50	Federal	520
**South East (7 medical schools)**	College of Health Sciences, Abia State University Uturu, Abia State	120	State	
	College of Medicine, University of Nigeria Enugu Campus, Enugu State	180	Federal	500
	College of Health Sciences, Nnamdi Azikiwe University Nnewi, Anambra State	100	Federal	368
	College of Medicine, Enugu State University of Science and Technology, Enugu, Enugu State	50	State	
	College of Medicine, Imo State University Owerri, Imo State	50	State	
	College of Health Sciences, Ebonyi State University Abakaliki, Ebonyi State	100	State	
	College of Health Sciences, Odumegwu Ojukwu University, Uli Anambra State	50	State	
**South South (9 medical schools)**	College of Health Sciences, University of Uyo, Akwa Ibom	50	Federal	499
	College of Medical Sciences, University of Calabar, Cross—Rivers State	100	Federal	610
	College of Health Sciences, Delta State University, Abraka, Delta State	50	State	250
	College of Medical Sciences, University of Benin, Benin-City, Edo State	150	Federal	701
	College of Health Sciences, Igbinedion University Okada, Edo State	75	Private	600
	College of Medicine, Ambrose Alli University Ekpoma, Edo State	50	State	
	College of Health Sciences, Madonna University Elele, Rivers State	50	Private	250
	College of Health Sciences, University of Port- Harcourt, Rivers State	150	Federal	782
	College of Health Sciences, Niger Delta University, Wilberforce Island, Bayelsa State	50	Federal	148
**South West (10 medical schools)**	College of Health Sciences, Afe Babalola University Ado-Ekiti, Ekiti State	100	Private	400
	College of Medicine, Ekiti State University, Ado-Ekiti	50	State	300
	College of Medicine, University of Lagos, Idi-Araba, Lagos State	150	Federal	761
	College of Medicine, Lagos State University Ikeja, Lagos State	100	State	
	College of Health Sciences, Olabisi Onabanjo University Ago Iwoye, Ogun Sate	75	State	
	College of Health Sciences, Obafemi Awolowo University Ile-Ife, Osun State	100	Federal	842
	College of Medicine, University of Ibadan, Oyo State	180	Federal	1,000
	College of Health Sciences, Ladoke Akintola University of Technology, Ogbomoso, Osun State	75	State	200
	College of Health Sciences, Bowen University, Iwo, Osun State	50	Private	400
	College of Health Sciences, Babcock University, Ilisham-Remo, Ogun State	50	Private	
**TOTAL QUOTA**	FOR ALL MEDICAL SCHOOLS ACROSS NIGERIA	**3,530**		

*MDCN* Medical and Dental Council of Nigeria

^a^Approved quota for medical and dental schools in Nigeria 2021 [20]

### Study setting

Medical training in Nigeria is a 6-year endeavour, typically with 1 year of preliminary basic sciences including physics, chemistry, and biology, about 2 years of pre-clinical training in anatomy, physiology, biochemistry, and other basic medical sciences, followed by 3 years of clinical training with clerkship rotations in surgery, internal medicine, obstetrics and gynaecology, paediatrics, and other sub-specialties [10]. Learning assessments occur through a variety of methods including oral and *viva-voce* examinations, multiple choice 1-in-5 or true or false questions, long case and short case clinical examinations, and a variety of Objective Structured Clinical Examinations depending on the medical school. Successful candidates are given a temporary practicing license upon graduation, however a 1-year supervised housemanship (internship) is a requisite for permanent licensing. Private medical schools are owned by missions, religious organizations and individuals and can be for-profit or not-for-profit institutions [21]. Public state organizations are either state government-owned, or federal government-owned [12]. Federal public institutions are largely the earliest established medical schools, state public medical schools are generally the second generation, and private medical schools are a relatively more recent addition to the medical schools in Nigeria [12].

Based on the MDCN national graduation quota of 3,530 students, [20] using the formula for sample size of cross-sectional studies, [22] we calculated a representative sample size of 349 recent graduates permitting a 5% margin of error and a 95% confidence level. To attain the required sample size, purposive sampling of recent graduates from one fully accredited Federal, State, and private medical school in each of the six geopolitical zones of Nigeria. Three zones did not have private medical schools. Although convenience sampling was used, spread in geography and type of institution was deliberate. Only medical doctors from the recent graduating class of the 15 selected accredited Nigerian universities who consented to the survey were included. Non-consenting individuals and those from non-accredited medical schools were excluded.

### Data collection tool

A face-validated, semi-structured, four-section, 35-multiple choice, Likert scale, and open-ended, online e-survey questionnaire was utilised (Additional file 1: Appendix 1) based on the Checklist for Reporting Results of Internet E-Surveys [23]. The survey was developed by a panel comprising experienced Nigerian and international surgical educators, and a recent medical graduate in an iterative process based on peer-reviewed literature and six focus group discussions with graduates. The procedures selected for inclusion in the finalised survey were based on relevant bedside procedures, procedures listed as necessary for primary and secondary care in the Lancet’s Disease Control Priorities Third Edition, [3] and three recognized ‘Bellwether’ procedures which serve as markers of access to surgical care (caesarean section, management of open fractures, and laparotomy) [24].

The e-survey was focused on self-reported number of procedures performed and confidence levels while performing the same procedures upon graduation using a 6-point confidence rating Likert scale. A score of 0 was “not at all confident” while 5 was “completely confident”. It was hosted on Google forms (Google, USA) and piloted for usability and technical functionality by 36 respondents from 15 medical schools, and these were excluded from final analysis.

The voluntary e-survey was distributed on class WhatsApp groups (WhatsApp USA) by a network of representatives who graduated from the selected institutions. WhatsApp was selected as it is a key communication platform used by recently graduated student groups. The survey was not openly distributed, and mandatory screening questions (year of graduation and school) were inserted to identify ineligible respondents. The maximum period that doctors must have left medical school was specified as 2019. As part of the consent process, respondents were introduced to the investigators and the purpose of the survey, told how data would be stored and utilised, in addition to the estimated length of the survey. Informed consent was obtained from each survey respondent on the opening page of the electronic questionnaire. No personal information was collected or stored, and access to data was limited to trained investigators. No incentives were offered to respondents. Data was collected from November 26, 2021, to January 9, 2022. Within this period, the survey distribution team met weekly to evaluate responses and to strategize for more effective dissemination. Log file analysis was used to screen for duplicate entries and atypical time stamps. Only completed questionnaires were analysed. Data was stored on an encrypted platform (Google Drive), and analysed on password-protected, encrypted systems to protect from unauthorised access.

#### Ethical clearance and funding

The study was approved by the Jos University Teaching Hospital ethics review committee (JUTH/DCS/IREC/127/XXX/2678). No funding was received for the survey.

### Statistical analysis

Data analysis was done on Microsoft Excel, R version 4.1.1. and JASP Software. Descriptive statistics (including simple percentages and means) were used to describe self-reported frequency of procedure performance. Confidence was analysed based on Likert scale of confidence interval and displayed in tables. Subgroup analysis of confidence by type and location of the institution was also performed using Fisher’s exact test. Using Fisher’s exact test, the number of procedures performed prior to graduation was compared by type of institution. For each procedure, Fisher’s exact test was also used to compare graduates that were confident with those that were not confident. A *p*-value of < 0.005 was considered significant.

**Fig. 2:**
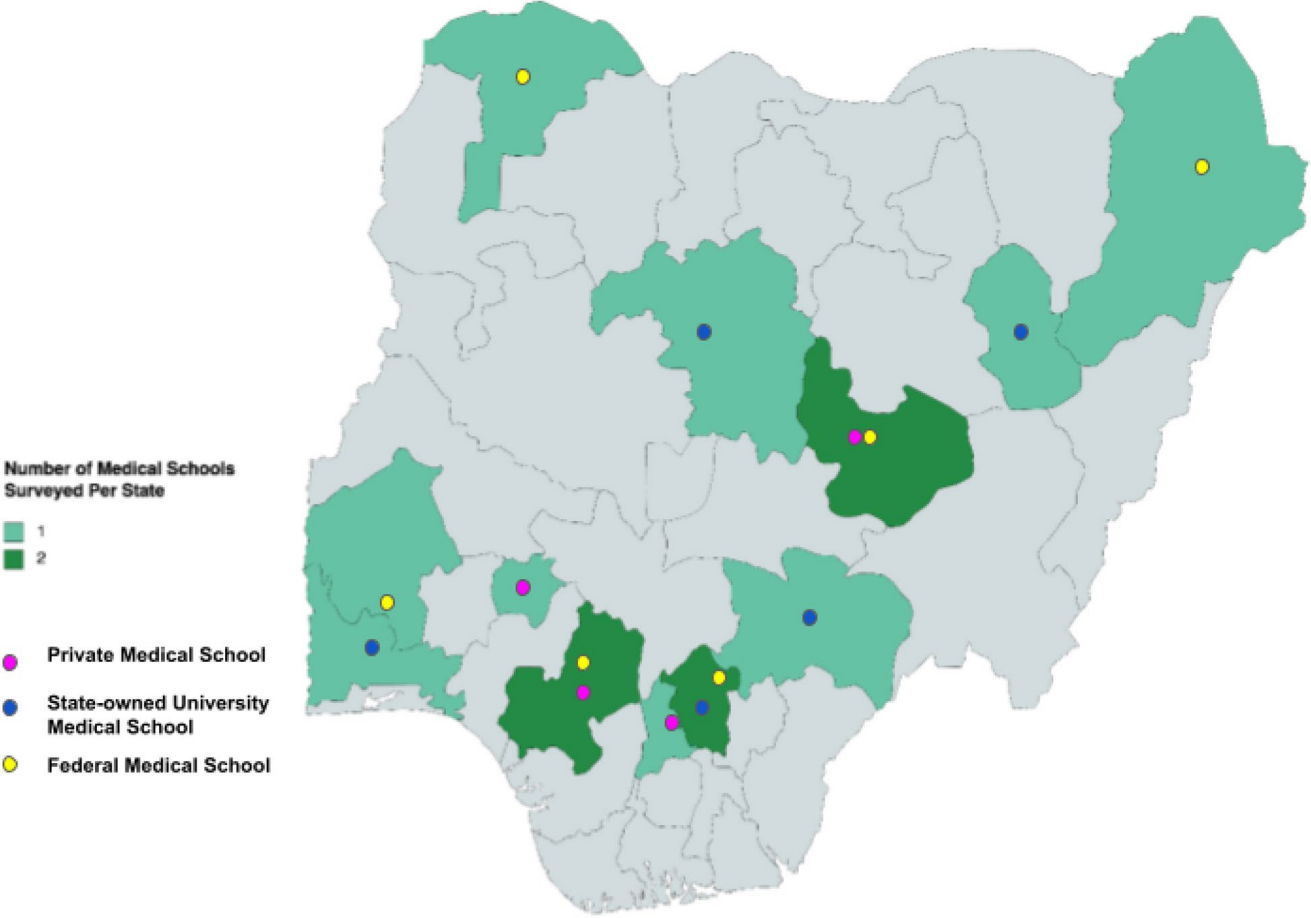
Location of medical schools that participants graduated from

We calculated and compared cumulative confidence scores by adding the confidence levels for each of the 15 procedures (ranging from 0 to 5 for each). This resulted in a cumulative confidence score range of 0–60. Qualitative analysis of free text recommendations to improve exposure to procedural skills in Nigerian medical schools as suggested by recent medical graduates was done using the constant comparative method in grounded theory. A series of online consensus meetings of 16 recent medical graduates from 15 Nigerian medical schools (Fig. 2) were held in which discussion of results, review of these free responses, and further comments were received to add depth to the recommendations.

## Results

There was a total of 449 responses out of 1,555 recent medical graduates from the selected medical schools, with a survey response rate of 29%. Response rate did not differ significantly for public federal (27%), public state (32%), or private institutions (34%), or by location (North East 16%, North West 28%, North Central 40%, South East 35%, South South 21%, South West 30%). The completion rate for respondents was 100%. The male-to-female ratio of respondents was 2:1, and most respondents (*n* = 263, 58.6%) were from public Federal medical schools (Table 2). Most respondents (*n* = 231, 51.4%) graduated in 2021, however, 151 (33.6%) graduated in 2020, while 67 (14.9%) graduated in 2019.

**Table 2:** Sociodemographics of Participants

Sociodemographic characteristics *N* = 449	n (%)
**Sex**	
Female	152 (33.9%)
Male	296 (65.9%)
Prefer not to say	1 (0.2%)
**Type of Institution attended**	
Public Federal medical schools	263 (58.6%)
Public State medical schools	118 (26.3%)
Private medical schools	68 (15.1%)
**Location (by national geo-political zone)**	
North-Central	109 (24.3%)
North-East	34 (7.6%)
North-West	58 (12.9%)
South-East	81 (18.0%)
South-West	114 (25.4%)
South-South	53 (11.8%)
**Medical School**	
University of Jos	64 (14.3%)
University of Ibadan	61 (13.6%)
University of Nigeria	55 (12.2%)
Usman Danfodio University	41 (9.1%)
Onabisi Onabanjo University	27 (6.0%)
Afe Babalola University Ado Ekiti	26 (5.8%)
Bingham University	26 (5.8%)
Enugu State University	26 (5.8%)
University of Benin	23 (5.1%)
Benue State University	19 (4.2%)
University of Maiduguri	19 (4.2%)
Kaduna State University	17 (3.8%)
Madonna University	16 (3.6%)
Gombe State University	15 (3.3%)
Ambrose Alli University	14 (3.1%)

### Exposure to clinical procedures and surgical experience prior to graduation

Table 3 shows the percentage of medical graduates in our sample who have had no experience independently performing procedures versus those with the most experience in performing procedures. The most experience was in securing peripheral access (104; 23%) while the least experience was in Bellwether procedures and other surgical interventions, World Bank Disease Control Priorities-3 Primary facility level surgical procedures.

**Table 3:** Recent graduates’ exposure to procedures prior to graduation- no experience versus most experience

Procedure Cluster and Procedure	Graduates with no experience at all in performing procedure n (%)	Graduates with most experience (> 10) n (%)
Bedside Procedures		
Securing peripheral intravenous access	83 (18%)	104 (23%)
Urethral catheterization	104 (23%)	73 (16%)
Control of haemorrhage	236 (53%)	15 (3%)
Incision and drainage of abscesses	323 (72%)	5 (1%)
Essential Obstetric Procedures		
Spontaneous vaginal delivery	253 (56%)	29 (6%)
Uterine evacuation	238 (53%)	6 (1%)
World Bank Disease Control Priorities-3 Primary facility level surgical procedures		
Male circumcision	400 (89%)	1 (0.2%)
Wound suturing	279 (62%)	13 (3%)
Hydrocelectomy	427 (95%)	0 (0%)
Debridement	269 (60%)	16 (4%)
Bellwether procedures and other surgical interventions		
Caesarean section	418 (93%)	1 (0.2%)
Laparotomy	423 (94%)	0 (0%)
Reduction of closed fractures	367 (82%)	2 (0.4%)
Initial management of open fractures	357 (80%)	0 (0%)
Closed thoracostomy tube insertion	382 (85%)	0 (0%)

There were significantly more catheterizations performed by learners in state and private medical schools than in federal schools (*p* < 0.05), and more circumcisions by those from state medical schools than private or federal medical schools (*p* = 0.028) (Additional file 1: Appendix II). However, more debridement and wound dressings were performed by learners who graduated from private institutions (*p* = 0.041) (Additional file 1: Appendix II). The highest median number of procedures were performed by students who graduated from State-owned medical schools (5 venous access, 5 catheterizations, 1 wound suturing), followed by private medical schools (4 venous access, 2.5 catheterizations, 1 debridement) (Additional file 1: Appendix III). Students graduating from Federal medical training institutions had the lowest median performance of procedures either under supervision or independently (Additional file 1: Appendix III). Learners from institutions in Northern Nigeria- specifically the North-East and North-West- performed the highest median number of procedures prior to graduation (Additional file 1: Appendix IV).

### Self-reported procedural confidence

**Fig. 3:**
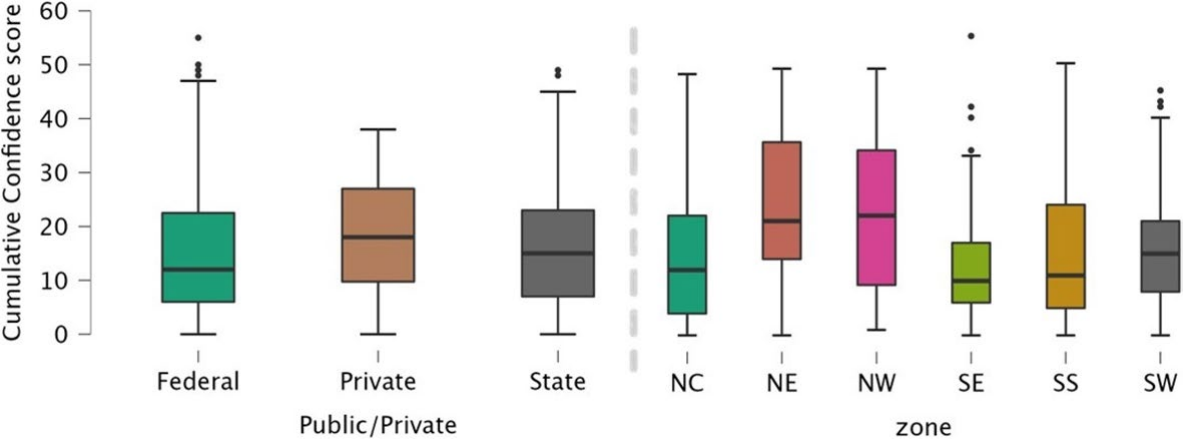
Cumulative Confidence Score by type of medical school, and by Geopolitical Zone. (NC- North Central; NE- North East; NW- North West, SE-South East, SS- South South; SW-South West)

Box plots of cumulative confidence scores showed that doctors who finished from private institutions were most confident in their ability to carry out procedures after graduation, followed by State and then Federal institutions (Fig. 3). Confidence was higher in the North East and North West (Fig. 3). No cumulative confidence score by an institution or a geopolitical zone exceeded 25/60. Graduates were most confident in urethral catheterization, and least confident in laparotomy (Table 4). Males were more confident overall than females in management of open fractures (X^2^ = 3.9; *p* = 0.048), and suturing lacerations (X^2^ = 6.9; *p* = 0.009) upon graduation (Additional file 1: Appendix V).

**Table 4:** Confidence ranking of procedures

Procedure	Frequency	Percentage (%)	Confidence Ranking/15
Urethral catheterization			1
Confident	253	56.347	
Not confident	196	43.653	
Intravenous Access			2
Confident	231	51.448	
Not confident	218	48.552	
Control of haemorrhage			3
Confident	141	31.403	
Not confident	308	68.597	
Vaginal delivery			4
Confident	116	25.835	
Not confident	333	74.165	
Suturing of lacerations			5
Confident	100	22.272	
Not confident	349	77.728	
Wound Debridement			6
Confident	99	22.049	
Not confident	350	77.951	
Incision and drainage			7
Confident	74	16.481	
Not confident	375	83.519	
Uterine Evacuation			8
Confident	50	11.136	
Not confident	399	88.864	
Management of open fractures			9
Confident	18	4.009	
Not confident	431	95.991	
Chest tube insertion			10
Confident	18	4.009	
Not confident	431	95.991	
Reduction of closed fractures			11
Confident	17	3.786	
Not confident	432	96.214	
Circumcision			12
Confident	14	3.118	
Not confident	435	96.882	
Caesarean section			13
Confident	13	2.895	
Not confident	435	96.882	
Hydrocelectomy			14
Confident	4	0.891	
Not confident	445	99.109	
Laparotomy			15
Confident	2	0.445	
Not confident	447	99.555	

Recommendations by recent graduates on how to improve medical students’ exposure to procedures are as seen in Table 5. Their recommendations ranged from provision of opportunities for students to independently perform procedures, improving student monitoring processes, the use of simulation and technology, creating smaller learner groups, increasing the length of clinical postings, introducing rural postings, and addressing bullying by trainers in the clinical space among others.

**Table 5:** Suggestions by recent medical school graduates for improved exposure to procedures and improved confidence performing procedures

**Theme**	**n**	**%**	
**Provide Opportunity:** Surgeons and residents to create more opportunities for students to perform independent procedures	148	59%	“…Then, in later years of clinicals, senior doctors should, with adequate enthusiasm, allow students to perform them, be it assisted or unassisted.”… Respondent (R) 77 “Our teachers should restructure the lectures in final year and make medical students to become more pragmatic and daring at the final level of graduation. Secondly house job experience should be more dedicated for doctors to learn practical medicine and not just for useless errand purposes. As I speak I'm yet to do a lot those surgical interventions even post house job.” R179
**Student Monitoring:** Faculty to actively monitor student case records and procedure logs and raise minimum standards for graduation	29	11%	“Requirements to sit for professional examinations should be improved a bit. Especially when it comes to log books, students should perform more procedures than assisting or even observing procedures before they can sit for examinations.”… 25 A logbook for the aforementioned procedures should be made available for the students to observe and perform.49
**Use Simulation:** Use of procedural simulation-based learning to enhance procedural learning	18	7%	“I suggest that a medical student should, right on time, be exposed to all of these procedures by first using dummies [manikins] in place of humans, at least at the first year in the clinicals and/or last year of pre-clinical.”
**Create Smaller Learner Groups**: Trainers to divide learners up into smaller student groups for maximal trainee attention, and better exposure to procedures	15	6%	“Students should be divided into small sub-groups in such a way that their number would not be a hindering factor for their learning and to also enable the supervisor to have good control and monitoring of their activities so as to make sure every student have performed the procedure.”
**Improve Attitude of Trainers:** Trainers to apply a more constructive and less critical approach to correcting students while learning procedures	14	6%	“Most doctors in teaching hospitals are mostly condescending while teaching. The environment is so tensed up, and they are quick to insult you for not grasping quickly. They forget that Rome was not built in a day. They should improve on their mannerism and students will desire to sleep in the theatre and accidents and emergency room each day.”
**Extend surgical and procedural postings**: Faculty to increase students exposure time to procedural skills	5	2%	“By making their hospital postings longer…” 46 Time for clinical posting should be increased.64 “Increase the duration of clinical activities” 86
**Direct senior consultant supervision:** More senior trainers (consultants) should supervise medical students directly	4	2%	Medical education should focus more in practical skills not just theories. Each posting should have a consultant set aside basically to teach medical student these basic medical skills. 176
**Vertical integration:** Graded exposure to clinical procedures before clinical levels	3	1%	“Practice makes perfect; the more procedures done, the higher the confidence. [We need] graded clinical skills exposure even before the clinical level.” 11
**Individualise training:** One-on-one mentoring in procedures	4	1%	“The procedure practice should be scheduled per students to allow for better distribution of procedures, instead of the 'survival of the fittest' approach to sign log books, where some students are favoured above others in getting practice”… 151 Theatres should be "theatres" where students can actually see what is being done and can follow the process taking place A student should be attached to a house officer for a period of one week at least where all he/she is expected to do is to work alongside the HO, I believe that would be quite the eye opening experience for students 91
**Procedure postings:** Allot postings strictly for procedures	4	1%	“There should be procedure posting, even if it's just a week of such activities.”… 30 A posting without lectures dedicated to these procedures 136
**Rural District Level Hospital Postings:** Post students to rural areas for more hands-on experience	3	1%	“Medical students should be posted to rural areas with a senior colleague for field experience, where they'll be required PERFORM 3–5 of these procedures before being signed out.”… 57 “Allow medical students do clinical posting 6 months in a general hospital.”..122
**Others: Increase number of clinical faculty, Motivate students, Leverage technology, and Improve facilities**	5	1%	“More staff clinical lecturers should be employed as the few don't have much time to carry the large number of medical students under their supervisions.” 147 “Medical students are overwhelmed with stresses so much that putting extra effort into engaging in procedural activities seem like more stress. A better quality of life and an honest motivation/incentive would suffice.”…37 “Use of screens/projectors to watch surgeries since the theatre shouldn't be crowded.” 157 “[Provide] Better [learning] facilities”. 203

## Discussion

Our findings suggest that exposure to procedural skills is generally low during undergraduate medical training in Nigeria, regardless of the type or location of the medical school attended. This holds true for both bedside procedures and operating room interventions. A previous study in South-South Nigeria also suggests that there is a need to improve medical students' exposure to practical skills. Upon graduation from a medical school in Port Harcourt, Nigeria, only 14 students in a class of 84 respondents (16.7%) had inserted more than 10 intravenous cannulae, while about half had never inserted a urethral catheter while in training [9]. Indeed, this deficit seems to be the case globally, as a recent systematic review showed a relatively high level of inexperience among new medical doctors in several countries. The greatest deficits in medical student exposure to procedural skills were in Iran, Nigeria, USA, then Bahrain, Kuwait, Oman, Qatar, Saudi Arabia, the United Arab Emirates, Egypt, and Ireland, followed by New Zealand and South Africa, with the least deficits identified in England [25].

In a system like Nigeria’s, where graduates from medical school are licensed to practice in district hospitals and provide basic medical and surgical care, this low exposure is particularly detrimental to patient safety. In this study, operative surgical exposure was found to be significantly lower than exposure to bedside procedures among medical students. Huo et al. reported generally low exposure to surgical procedures in second-year medical students and also noted a significantly lower exposure to more invasive procedures [26]. The low-risk, high-volume nature of bedside procedures relative to operating room procedures explains the higher frequency of performance of procedures like securing intravenous access or catheterization as compared with abdominal laparotomy [27]. Possible reason for the poor exposure has been described by educational scholars as the “neglect of the medical student” [28, 29]. In tertiary teaching centres, the competition for procedural exposure is high. Resident doctors and more senior trainees receive precedence for practical surgical exposure, at the expense of medical students. In the event that an attending or consultant surgeon does not personally do the procedure, the medical student will be considered only after the senior or junior resident, and house officers or interns who are also competing for these exposures [30]. The relatively large number of medical students in some training centres is contributory, as clinical instructors are overburdened with a high student-to-clinical tutor ratio [20, 30]. Large student quotas in hospitals with capped patient load also hinder student exposure, as they may watch procedures, but never perform them. In response to this, recent medical graduates in Nigeria recommend the creation of more opportunities for supervised practice prior to graduation including an extension of surgical rotation periods, one-on-one mentoring, and the creation of smaller learner groups. Improved exposure to simulation-based learning in Nigerian medical schools can also result in better procedural exposure [10, 31, 32].

Exposure to procedures was maximal in the North East and North West geopolitical zones. This may not be unrelated to the paucity of tertiary training centres in these regions on the backdrop of a teeming population. North West Nigeria is the most populated region, hosting an estimated population over 46 million with only three fully accredited medical schools. Similarly, despite a population of approximately 30 million, North Eastern Nigeria has only two accredited medical schools [20]. The resulting low medical doctor density causes medical students and recent graduates to assume more practical roles, as opposed to other regions such as the South Eastern region boasting of seven teaching centres, and a population less than 20 million [20]. This might also explain the relatively lower response rate of 19% from graduates in this region, as they may have been under more time pressure from direct patient care during their medical practice in this particular geopolitical region. Despite the fact that both of these northern regions account for over 35% of Nigeria’s overall population, they jointly account for only 12% of Nigeria’s doctors and less than a fourth of the nurses [2, 33]. This also implies an increased workload on the limited workforce which may extend to the medical students. The waves of insurgency and banditry which have plagued these two regions have resulted in displacement and increased predisposition to illnesses and injury. For example, the Boko-haram insurgency at its peak displaced millions of citizens to camps where many life-threatening diseases were observed to be rampant, cholera outbreak inclusive outbreaks [34]. The contribution of multiple adverse social determinants of health, including higher levels of poverty and illiteracy, in addition to a limited number and spread of healthcare facilities, results in a high burden of neglected surgical conditions being seen at the few available facilities [35–37]. This increased patient burden provides opportunities for students of medical colleges to experience more hands-on training. The reason for the significant difference between male and female confidence in operative orthopaedic and general surgery procedures is not particularly clear. Previous studies suggest that it can be attributable to the “male-surgeon” stereotype, the likelihood of males to aggressively contend for procedures, and the likelihood of males choosing a surgical elective course [27].

Graduates from private universities were more confident in their ability to perform procedures as compared to those who finished from other universities. While it has been established that a student's self-esteem is directly proportional to scholastic performance, “toxic practice” may play a role in reducing learner esteem despite high practical exposure [38]. Senior colleagues and trainers often belittle and harass faltering medical students to improve performance in Nigeria public medical schools, in a reflection of a colonial strategy. Olasoji reported an 85% prevalence of “toxic practice” in University of Maiduguri with more than half of the students claiming the teaching method affected them negatively [39]. With depression rates of over 30%, and psychoactive substance use in almost half of these students, as identified by a systematic review, high levels of psychological stress and poor mental health among Nigerian medical students can be contributory [10]. Up to 6% of respondents suggested that addressing “toxic practice” would improve exposure. One graduate from North Central Nigeria stated, “Most doctors in teaching hospitals are mostly condescending while teaching. The environment is so tensed up, and they are quick to insult you for not grasping quickly. They forget that Rome was not built in a day. They should improve on their mannerism and students will desire to sleep [stay for long periods] in the [operating] theatre and accidents and emergency room each day [learning and performing clinical procedures].” Another stated, “If I had been encouraged with a senior colleague beside me [and] assisting me, who wouldn't insult or make me feel useless, I think I would have done better.” Another respondent from the South West stated, “They should have allowed us to assist more and not berate us for making mistakes since were trying to learn.” Oku and colleagues found that over a third of Nigerian medical students that were surveyed in one institution experienced abuse, bullying, and mistreatment, up to 38% claiming that this occurred weekly [39]. This creates a significant psychological and physical barrier to performing procedures. A global systematic review showed that “toxic practice” reduces confidence of up to 27% of medical trainees, and results in considerations of discontinuing training in up to 36% [38]. Addressing this is vital to improving medical student exposures to, and confidence in performing procedures [38].

The survey recommendations suggest that improved facilities may result in improved exposure. For example, most teaching hospitals and universities in Nigeria have divergent administrative teams and receive parallel funding, therefore, disagreements on budget and spending may arise that disadvantage the medical students. Students often have to purchase their own consumables including examination gloves, to perform procedures for training and research when disputes arise when conflicts of interest arise between medical schools and teaching hospitals [10].

As recommended by learners, there is evidence that simulation-based learning and leveraging technology enhances procedural skill and is possible in low resource contexts [31, 32, 40]. Rural postings, smaller learning groups, and close proctoring also have the potential of improving learner confidence and exposure to practical procedures. Extension of training, as recommended by 2% of the learners would need a readjustment of current Nigerian Medical School curricula and timelines [10]. In addition, internship must be considered as an opportunity to extend practical training [13] An example of a recommendation that highlights this potential is as shown, “Secondly, house job (internship) experience should be more dedicated for doctors to learn practical medicine and not just for errand purposes. As I speak, I'm yet to do a lot those surgical interventions…”.

### Limitations of the study

Confidence must be correlated with the quality of performance for a more robust evaluation. Competence is better assessed by a trainer as self-adjudged competence or confidence is not always an accurate marker. This could have been objectively assessed if the competence level was reported from a validated logbook while students were in training. Some learners may report high levels of confidence regardless of their capacity to perform a procedure [41]. Intra-operative observations and Objective Structured Assessment of Technical Skills of recent graduates would also have addressed this nuance. Students who can afford medical education at a costly private institution often come from a more privileged background. Their financial pedigree may also lead to higher overall confidence which may be reflected in our results [42]. Further qualitative studies will be necessary to explore this in detail. This study also has the potential for selection bias if some doctors are less likely to join their class WhatsApp groups or do not have access to the internet for any reason. However, across Nigeria, there is widespread use of WhatsApp for communication, and students primarily rally their classes platform [43]. We were, however, not able to ascertain whether there were any differences in social media use or creation of alumni groups among subgroups in the sample. In addition, there is the potential for recall bias, however, majority of the participants left medical school within the previous year. The nationwide sampling of medical graduates made possible by extensive collaboration between medical graduates were significant strengths of this study.

### Recommendations

The study group recommends the following:

1. Learners in Nigerian medical schools should actively seek opportunities to perform procedures especially at the emergency room and during call duty hours. They should leverage technology, gamification, and artificial intelligence to maximize exposure to procedures.
2. Faculty should provide opportunities for learners to perform procedures in a non-toxic learning environment and monitor students’ procedural skill development in a formative manner. Faculty should aim at small learner groups and give constructive, individualized feedback to medical students.
3. Medical training institutions should perform vertical integration of procedural skills into their curricula and integrate simulation and practical mentoring programmes. Institutions should also consider extending surgical and procedural rotations, and increasing facilities, including simulation laboratories, operating room extensions with monitors. Findings from this study should be considered during the review of the national medical curriculum to emphasise procedural skills and exposure [10].
4. State and Federal governments should consider decentralized models of training. The model of medical education anchored solely in urban, tertiary hospital training sites must be reconsidered, and rural district hospitals postings should be recommended. Most rural centres do not have sufficient workforce and are a potential location for more hands-on experience for students.

## Conclusion

In conclusion, our study highlights low exposure to procedural skills during undergraduate medical training in Nigeria, regardless of the type or location of the medical school attended. This deficit extends to both bedside procedures and operating room interventions, with operative exposure being particularly low. Our findings emphasize the need for comprehensive reforms in medical education to enhance procedural exposure and skill development. This will ultimately lead to improved patient care and better outcomes in Nigeria's healthcare system. The findings are consistent with previous regional studies and global trends, and emphasize the urgent need to improve practical skill exposure among medical students in Nigeria, and indeed, wordwide.

The marginalization of medical students within tertiary teaching centers, the challenge of limited resources, high student-to-clinical tutor ratios, and the disruptive effects of strikes in federal hospitals contribute to the inadequate procedural exposure. Regional disparities in exposure to procedures are likely influenced by population density, limited healthcare facilities, and the impact of insurgency and banditry. Learners, faculty, medical school and teaching hospital administration, and government all have a role to play in addressing this challenge through monitoring and evaluation, innovation and the creation of new models of training.

### Supplementary Information

**Additional file 1:** **Appendix I.** A copy of the survey. **Appendix II.** Number of procedures performed prior to graduation from medical school as lead, under supervision, by type of institution*. **Appendix III.** Median number of procedures performed prior to graduation, by type of medical school. **Appendix IV.** Number of Procedures performed prior to graduation by medical school location (geopolitical zone). **Appendix V. **Overall self-reported confidence score in performing procedures.

## Data Availability

The datasets used and/or analysed during the current study are available from the corresponding author on reasonable request.

## Abbreviations

MDCN: Medical and Dental Council of Nigeria
LMICs: Low- and Middle-Income Countries
