# Supplementary material for: A national perspective on exposure to essential surgical procedures among medical trainees in Nigeria: a cross-sectional survey and recommendations
Source: BMC Med Educ. 2023 Nov 30;23:913. doi: 10.1186/s12909-023-04847-4 (PMC10691202; doi:10.1186/s12909-023-04847-4)
Supplement: Supplementary file 1 — Additional file 1: Appendix I. A copy of the survey. Appendix II. Number of procedures performed prior to graduation from medical school as lead, under supervision, by type of institution*. Appendix III. Median number of procedures performed prior to graduation, by type of medical school. Appendix IV. Number of Procedures performed prior to graduation by medical school location (geopolitical zone). Appendix V. Overall self-reported confidence score in performing procedures. [file 12909_2023_4847_MOESM1_ESM.docx]

**A National Perspective on Exposure to Essential Surgical Procedures among Medical Trainees in Nigeria: A Cross-Sectional Survey and Recommendations**

**List of Appendices**

Appendix I: A copy of the survey

Appendix II: Number of procedures performed prior to graduation from medical school as lead, under supervision, by type of institution*

Appendix III: Median number of procedures performed prior to graduation, by type of medical school

Appendix IV: Number of Procedures performed prior to graduation by medical school location (geopolitical zone)

Appendix V: Overall self-reported confidence score in performing procedures

**Appendix I: Survey**

**An assessment of procedural technical skills among recent Nigerian medical graduates (University's Name)**

I am Dr. Barnabas Alayande, a General Surgeon/Global Surgery Fellow affiliated to Jos University Teaching Hospital. This survey is to assess the experience and confidence of recent medical graduates in Nigeria in carrying out basic procedural technical skills. This survey will take only 5 minutes.

Participation is free and voluntary, and refusal to participate will not infringe on participants' right. All responses are confidential and will be treated as such. This work will serve as a basis for recommendations for the improvement of Nigerian medical education. Please email barnabasalayande@yahoo.com with any questions or concerns. Thank you!

Kindly indicate your consent by continuing with the survey.

NB: This assessment focuses on experience and confidence *as at time of graduation* and not necessarily your current.

* Indicates required question

1. Year of completion of medical school * *Mark only one .* 2021 2020 2019

2. Sex *Mark only one .* Female Male

**3. Number of Procedures That You Performed (not assisted!) in Medical School.**

This is to assess the experience level and participation of recent medical graduates in the underlisted procedures as at the TIME OF COMPLETION OF MEDICAL SCHOOL. Kindly identify only the number of procedures you carried out either independently or as the lead under supervision.

**3a. Intravenous access** (setting of line) performed as at graduation **Mark only one .*

0, 1, 2, 3, 4, 5, 6, 7, 8, 9, 10 Select 10 for > 10

**3b. Urethral catheterization** performed as at graduation **Mark only one .*

0, 1, 2, 3, 4, 5, 6, 7, 8, 9, 10 Select 10 for > 10

**3c. Vaginal delivery** performed as at graduation **Mark only one .*

0, 1, 2, 3, 4, 5, 6, 7, 8, 9, 10 Select 10 for > 10

**3d. Uterine evacuation** performed as at graduation **Mark only one .*

0, 1, 2, 3, 4, 5, 6, 7, 8, 9, 10 Select 10 for > 10

**3e. Circumcision** performed as at graduation **Mark only one .*

0, 1, 2, 3, 4, 5, 6, 7, 8, 9, 10 Select 10 for > 10

**3f. Hydrocoele surgery** performed as at graduation **Mark only one .*

0, 1, 2, 3, 4, 5, 6, 7, 8, 9, 10 Select 10 for > 10

**3g. Incision and drainage** performed as at graduation **Mark only one .*

0, 1, 2, 3, 4, 5, 6, 7, 8, 9, 10 Select 10 for > 10

**3h. Wound suturing** performed as at graduation **Mark only one .*

0, 1, 2, 3, 4, 5, 6, 7, 8, 9, 10 Select 10 for > 10

**3i. Control of haemorrhage** with pressure dressings performed as at graduation **Mark only one .*

0, 1, 2, 3, 4, 5, 6, 7, 8, 9, 10 Select 10 for > 10

**3j. Debridement and dressing** of wounds performed as at graduation **Mark only one .*

0, 1, 2, 3, 4, 5, 6, 7, 8, 9, 10 Select 10 for > 10

**3k. Reduction of closed fractures** performed as at graduation **Mark only one .*

0, 1, 2, 3, 4, 5, 6, 7, 8, 9, 10 Select 10 for > 10

**3l. Management of open fractures** performed as at graduation **Mark only one .*

0, 1, 2, 3, 4, 5, 6, 7, 8, 9, 10 Select 10 for > 10

**3m. Chest tube insertion** performed as at graduation **Mark only one .*

**3n. Caesarean section** performed as at graduation **Mark only one .*

0, 1, 2, 3, 4, 5, 6, 7, 8, 9, 10 Select 10 for > 10

**3o. Laparotomy** performed as at graduation * *Mark only one .*

0, 1, 2, 3, 4, 5, 6, 7, 8, 9, 10 Select 10 for > 10

**4. Confidence level in your ability to carry out procedures** at the time of your graduation from medical school.

Select zero (0) for "not at all confident" and five (5) for "completely confident"

4a. How confident were you in **intravenous access** (setting of a peripheral line) on graduation? *Mark only one .*  Not at all confident 0, 1, 2, 3, 4, 5 Completely confident

4b. How confident were you in performing **urethral catheterization** on graduation? **Mark only one .*

Not at all confident 0, 1, 2, 3, 4, 5 Completely confident

4c. How confident were you in managing **normal labour** up till delivery on graduation? *Mark only one .*

Not at all confident 0, 1, 2, 3, 4, 5 Completely confident

4d. How confident were you in performing **uterine evacuation** on graduation? **Mark only one .*

Not at all confident 0, 1, 2, 3, 4, 5 Completely confident

4e. How confident were you in performing **circumcision** on graduation? **Mark only one*

Not at all confident 0, 1, 2, 3, 4, 5 Completely confident*.*

4f. How confident were you in performing **hydrocoele surgery** on graduation? **Mark only one .*

Not at all confident 0, 1, 2, 3, 4, 5 Completely confident

4g. How confident were you in performing **incision and drainage** on graduation? **Mark only one.*

Not at all confident 0, 1, 2, 3, 4, 5 Completely confident

4h. How confident were you in performing **wound suturing** on graduation? **Mark only one .*

Not at all confident 0, 1, 2, 3, 4, 5 Completely confident

4i. How confident were you in performing **control of haemorrhage** with pressure dressings on graduation? **Mark only one.* Not at all confident 0, 1, 2, 3, 4, 5 Completely confident

4j. How confident were you in performing **debridement and dressings** of wounds on graduation?

Not at all confident 0, 1, 2, 3, 4, 5 Completely confident

4k. How confident were you in performing **reduction of closed fractures** on graduation? *Mark only one .*

Not at all confident 0, 1, 2, 3, 4, 5 Completely confident

4l. How confident were you managing **open fractures** on graduation? **Mark only one .*

Not at all confident 0, 1, 2, 3, 4, 5 Completely confident

4m. How confident were you performing **chest tube insertion** on graduation? **Mark only one .*

Not at all confident 0, 1, 2, 3, 4, 5 Completely confident

4n. How confident were you performing **caesarean section** on graduation? **Mark only one .*

Not at all confident 0, 1, 2, 3, 4, 5 Completely confident

4o. How confident were you in doing **Laparotomy** on graduation? * *Mark only one .*

Not at all confident 0, 1, 2, 3, 4, 5 Completely confident

5. **Your recommendations**: What could have been done in medical school to improve your confidence in doing procedures? How can exposure of medical students to procedures be made more adequate?

_______________________________________________________________

_______________________________________________________________

_______________________________________________________________

_______________________________________________________________

_______________________________________________________________

**Appendix II: Number of procedures performed prior to graduation from medical school as lead, under supervision, by type of institution (using Fisher’s exact test)**

| Numbers of Procedures performed | None  n (%) | One to Five  n (%) | Six to Nine  n (%) | ≥ Ten  n (%) | X^2^ (Fishers exact test), p | Cramer's V |
| --- | --- | --- | --- | --- | --- | --- |
| Intravenous access |  |  |  |  | 13.751, 0.33 | 0.124 |
| Public Federal medical schools | 52 (20%) | 50 (19 %) | 137 (52%) | 50 (19%) |  |  |
| Public State medical schools | 19 (16%) | 43 (37%) | 18 (15%) | 38 (32%) |  |  |
| Private medical schools | 12 (18%) | 32 (48%) | 8 (12%) | 16 (24%) |  |  |
| Catheterization |  |  |  |  | 87.317, <0.05* | 0.312 |
| Public Federal medical schools | 69 (26%) | 137 (52%) | 28 (11%) | 29 (11%) |  |  |
| Public State medical schools | 13 (11%) | 42 (36%) | 16 (14%) | 47 (40%) |  |  |
| Private medical schools | 10 (15%) | 14 (21%) | 4 (6%) | 40 (59%) |  |  |
| Vaginal delivery |  |  |  |  | 17.055, 0.009* | 0.138 |
| Public Federal medical schools | 161 (61%) | 75 (29%) | 12 (5%) | 15 (6%) |  |  |
| Public State medical schools | 49 (42%) | 50 (42%) | 7 (6%) | 12 (10%) |  |  |
| Private medical schools | 43 (63%) | 22 (32%) | 1 (2%) | 2 (3%) |  |  |
| Uterine evacuation |  |  |  |  | 7.182, 0.304 | 0.089 |
| Public Federal medical schools | 206 (78%) | 49 (19%) | 4 (2%) | 4 (2%) |  |  |
| Public State medical schools | 79 (67%) | 35 (30%) | 2 (2%) | 2 (2%) |  |  |
| Private medical schools | 53 (78%) | 14 (21%) | 1 (1%) | 0 (0%) |  |  |
| Circumcision |  |  |  |  | 14.174, 0.028* | 0.126 |
| Public Federal medical schools | 244 (93%) | 18 (7%) | 0 (0%) | 1 (0%) |  |  |
| Public State medical schools | 96 (81%) | 21 (18%) | 1 (1%) | 0 (0%) |  |  |
| Private medical schools | 60 (88%) | 8 (12%) | 0 (0%) | 0 (0.00%) |  |  |
| Hydrocoele Surgery |  |  |  |  | 0.369, 0.832 | 0.029 |
| Public Federal medical schools | 251 (95%) | 12 (5%) | 0 (0%) | 0 (0%) |  |  |
| Public State medical schools | 111 (94%) | 7 (6%) | 0 (0%) | 0 (0%) |  |  |
| Private medical schools | 65 (96%) | 3 (4%) | 0 (0%) | 0 (0%) |  |  |
| Incision and Drainage |  |  |  |  | 5.361, 0.498 | 0.077 |
| Public Federal medical schools | 190 (72%) | 64 (24%) | 5 (2%) | 4 (2%) |  |  |
| Public State medical schools | 80 (68%) | 36 (31%) | 1 (1%) | 1 (1%) |  |  |
| Private medical schools | 53 (78%) | 13 (19%) | 2 (3%) | 0 (0%) |  |  |
| Wound suturing |  |  |  |  | 6.504, 0.369 | 0.085 |
| Public Federal medical schools | 173 (66%) | 80 (30%) | 4 (2%) | 6 (2%) |  |  |
| Public State medical schools | 65 (55%) | 45 (38%) | 2 (2%) | 6 (5%) |  |  |
| Private medical schools | 41 (60%) | 24 (35%) | 2 (3%) | 1 (1%) |  |  |
| Control of Haemorrhage |  |  |  |  | 5.740, 0.453 | 0.080 |
| Public Federal medical schools | 144 (55%) | 103 (39%) | 7 (3%) | 9 (3%) |  |  |
| Public State medical schools | 52 (44%) | 56 (47%) | 5 (4%) | 5 (4%) |  |  |
| Private medical schools | 40 (59%) | 25 (37%) | 2 (3%) | 1 (1%) |  |  |
| Debridement and Dressing |  |  |  |  | 13.107, 0.041* | 0.121 |
| Public Federal medical schools | 172 (65%) | 81 (31%) | 3 (1%) | 7 (3%) |  |  |
| Public State medical schools | 66 (56%) | 42 (36%) | 5 (4%) | 5 (4%) |  |  |
| Private medical schools | 31 (46%) | 30 (44%) | 3 (4%) | 4 (6%) |  |  |
| Reduction of Closed Fractures |  |  |  |  | 12.521, 0.051 | 0.118 |
| Public Federal medical schools | 224 (85%) | 37 (14%) | 0 (0%) | 2 (1%) |  |  |
| Public State medical schools | 92 (78%) | 26 (22%) | 0 (0%) | 0 (0%) |  |  |
| Private medical schools | 51 (75%) | 16 (24%) | 1 (1%) | 0 (0%) |  |  |
| Management of Open Fracture |  |  |  |  | 12.820, 0.012 | 0.119 |
| Public Federal medical schools | 220 (84%) | 39 (15%) | 4 (2%) | 0 (0%) |  |  |
| Public State medical schools | 84 (71%) | 34 (29%) | 0 (0%) | 0 (0%) |  |  |
| Private medical schools | 53 (78%) | 15 (22%) | 0 (0%) | 0 (0%) |  |  |
| Chest Tube Insertion |  |  |  |  | 2.076, 0.722 | 0.048 |
| Public Federal medical schools | 228 (87%) | 34 (13%) | 1 (0%) | 0 (0%) |  |  |
| Public State medical schools | 98 (83%) | 19 (16%) | 1 (1%) | 0 (0%) |  |  |
| Private medical schools | 56 (82%) | 12 (18%) | 0 (0%) | 0 (0%) |  |  |
| Caesarean Section |  |  |  |  | 4.299, 0.367 | 0.069 |
| Public Federal medical schools | 249 (95%) | 13 (5%) | 0 (0%) | 1 (0%) |  |  |
| Public State medical schools | 106 (90%) | 12 (10%) | 0 (0%) | 0 (0%) |  |  |
| Private medical schools | 63 (93%) | 5 (7%) | 0 (0%) | 0 (0%) |  |  |
| Laparotomy |  |  |  |  | 2.324, 0.313 | 0.072 |
| Public Federal medical schools | 252 (96%) | 11 (4%) | 0 (0%) | 0 (0%) |  |  |
| Public State medical schools | 109 (92%) | 9 (8%) | 0 (0%) | 0 (0%) |  |  |
| Private medical schools | 63 (93%) | 5 (7%) | 0 (0%) | 0 (0%) |  |  |

Only procedures performed as lead under supervision or as independent lead; Fisher’s exact test was used when cells contained figures <5; *=statistically significant

**Appendix III: Median number of procedures performed prior to graduation, by type of medical school**

|  | IV Access | Catheter-  ization | Vaginal delivery | Uterine evacuation | Circumcision | Hydrocelectomy | Incision and Drainage | Wound suturing | Haemorrhage Control | Debridement | Reduction of closed fractures | Open fracture management | Thoracostomy tube insertion | Caesarean section | Laparotomy |
| --- | --- | --- | --- | --- | --- | --- | --- | --- | --- | --- | --- | --- | --- | --- | --- |
| Number reported as performed* | 1992 | 1708 | 780 | 323 | 91 | 35 | 313 | 528 | 703 | 558 | 168 | 190 | 107 | 56 | 39 |
| Public Federal medical schools | 1034 | 832 | 411 | 178 | 39 | 20 | 187 | 262 | 383 | 261 | 90 | 101 | 57 | 30 | 18 |
| **Median** | 3 | 2 | 0 | 0 | 0 | 0 | 0 | 0 | 0 | 0 | 0 | 0 | 0 | 0 | 0 |
| Interquartile range | 6 | 5 | 2 | 0 | 0 | 0 | 1 | 1 | 2 | 1 | 0 | 0 | 0 | 0 | 0 |
| Lower quartile | 1 | 0 | 0 | 0 | 0 | 0 | 0 | 0 | 0 | 0 | 0 | 0 | 0 | 0 | 0 |
| Upper Quartile | 7 | 5 | 2 | 0 | 0 | 0 | 1 | 1 | 2 | 1 | 0 | 0 | 0 | 0 | 0 |
| Public State medical schools | 648 | 650 | 300 | 110 | 41 | 11 | 84 | 186 | 238 | 155 | 43 | 56 | 36 | 19 | 16 |
| **Median** | **5** | 5 | 1 | 0 | 0 | 0 | 0 | 0 | 1 | 0 | 0 | 0 | 0 | 0 | 0 |
| Interquartile range | 8 | 8 | 4 | 1 | 0 | 0 | 1 | 2 | 3 | 1 | 0 | 1 | 0 | 0 | 0 |
| Lower quartile | 2 | 2 | 0 | 0 | 0 | 0 | 0 | 0 | 0 | 0 | 0 | 0 | 0 | 0 | 0 |
| Upper quartile | 10 | 10 | 4 | 1 | 0 | 0 | 1 | 2 | 3 | 1 | 0 | 1 | 0 | 0 | 0 |
| Private medical schools | 310 | 226 | 69 | 35 | 11 | 4 | 42 | 80 | 82 | 142 | 35 | 33 | 14 | 7 | 5 |
| **Median** | **4** | 2.5 | 0 | 0 | 0 | 0 | 0 | 0 | 0 | 1 | 0 | 0 | 0 | 0 | 0 |
| Interquartile range | 8 | 5.25 | 1 | 0 | 0 | 0 | 0 | 1 | 2 | 3 | 0.25 | 0 | 0 | 0 | 0 |
| Lower quartile | 1 | 0 | 0 | 0 | 0 | 0 | 0 | 0 | 0 | 0 | 0 | 0 | 0 | 0 | 0 |
| Upper quartile | 9 | 5.25 | 1 | 0 | 0 | 0 | 0 | 1 | 2 | 3 | 0.25 | 0 | 0 | 0 | 0 |

*Performed refers to procedures done as lead surgeon either under supervision or independently

**Appendix IV: Number of Procedures performed prior to graduation by medical school location (geopolitical zone)**

|  | Intravenous access | Catheter-  ization | Vaginal delivery | Uterine evacuation | Circumcision | Hydrocelectomy | Incision and drainage | Wound suturing | Haemorrhage Control | Debridement | Reduction of closed fractures | Open fracture management | Chest tube insertion | Caesarean section | Laparotomy |
| --- | --- | --- | --- | --- | --- | --- | --- | --- | --- | --- | --- | --- | --- | --- | --- |
| Number reported as performed* across all sites | 1992 | 1708 | 780 | 323 | 91 | 35 | 313 | 528 | 703 | 558 | 168 | 190 | 107 | 56 | 39 |
| Total from North Central | 369 | 318 | 149 | 68 | 4 | 3 | 72 | 111 | 158 | 176 | 49 | 49 | 19 | 14 | 3 |
| **Median** | 2 | 2 | 0 | 0 | 0 | 0 | 0 | 0 | 1 | 0 | 0 | 0 | 0 | 0 | 0 |
| Interquartile range | 6 | 4 | 2 | 0 | 0 | 0 | 1 | 1 | 2 | 2 | 0 | 0 | 0 | 0 | 0 |
| Lower quartile | 0 | 0 | 0 | 0 | 0 | 0 | 0 | 0 | 0 | 0 | 0 | 0 | 0 | 0 | 0 |
| Upper Quartile | 6 | 4 | 2 | 0 | 0 | 0 | 1 | 1 | 2 | 2 | 0 | 0 | 0 | 0 | 0 |
| North East | 209 | 175 | 123 | 79 | 23 | 9 | 60 | 89 | 106 | 74 | 21 | 23 | 15 | 8 | 2 |
| **Median** | 7.5 | 5 | 4 | 1 | 0 | 0 | 0.5 | 1 | 2 | 1 | 0 | 0 | 0 | 0 | 0 |
| Interquartile range | 6 | 7.75 | 5.75 | 4 | 0 | 0 | 3 | 3 | 3 | 4 | 0 | 0.75 | 0.75 | 0.75 | 0 |
| Lower quartile | 4 | 2 | 0.25 | 0 | 0 | 0 | 0 | 0 | 1 | 0 | 0 | 0 | 0 | 0 | 0 |
| Upper Quartile | 10 | 9.75 | 6 | 4 | 0 | 0 | 3 | 3 | 4 | 4 | 0 | 0.75 | 0.75 | 0.75 | 0 |
| North West | 422 | 324 | 197 | 81 | 10 | 5 | 69 | 117 | 126 | 115 | 26 | 39 | 30 | 11 | 10 |
| **Median** | 10 | 6 | 1 | 0 | 0 | 0 | 0 | 1 | 1 | 1 | 0 | 0 | 0 | 0 | 0 |
| Interquartile range | 6.75 | 9 | 6.75 | 2.75 | 0 | 0 | 2 | 3 | 3.75 | 3 | 0 | 1 | 0.75 | 0 | 0 |
| Lower quartile | 3.25 | 1 | 0 | 0 | 0 | 0 | 0 | 0 | 0 | 0 | 0 | 0 | 0 | 0 | 0 |
| Upper Quartile | 10 | 10 | 6.75 | 2.75 | 0 | 0 | 2 | 3 | 3.75 | 3 | 0 | 1 | 0.75 | 0 | 0 |
| South East | 271 | 251 | 154 | 38 | 6 | 6 | 32 | 37 | 71 | 45 | 20 | 21 | 15 | 5 | 3 |
| **Median** | 2 | 2 | 0 | 0 | 0 | 0 | 0 | 0 | 0 | 0 | 0 | 0 | 0 | 0 | 0 |
| Interquartile range | 4 | 4 | 3 | 0 | 0 | 0 | 1 | 1 | 1 | 0 | 0 | 0 | 0 | 0 | 0 |
| Lower quartile | 1 | 0 | 0 | 0 | 0 | 0 | 0 | 0 | 0 | 0 | 0 | 0 | 0 | 0 | 0 |
| Upper Quartile | 5 | 4 | 3 | 0 | 0 | 0 | 1 | 1 | 1 | 0 | 0 | 0 | 0 | 0 | 0 |
| South South | 220 | 179 | 47 | 21 | 18 | 0 | 37 | 59 | 89 | 51 | 27 | 18 | 3 | 5 | 5 |
| **Median** | 3 | 2 | 0 | 0 | 0 | 0 | 0 | 0 | 0 | 0 | 0 | 0 | 0 | 0 | 0 |
| Interquartile range | 6 | 6 | 1 | 0 | 0 | 0 | 1 | 1 | 3 | 1 | 0 | 0 | 0 | 0 | 0 |
| Lower quartile | 1 | 0 | 0 | 0 | 0 | 0 | 0 | 0 | 0 | 0 | 0 | 0 | 0 | 0 | 0 |
| Upper Quartile | 7 | 6 | 1 | 0 | 0 | 0 | 1 | 1 | 3 | 1 | 0 | 0 | 0 | 0 | 0 |
| South West | 501 | 461 | 110 | 36 | 30 | 12 | 43 | 115 | 153 | 97 | 25 | 40 | 25 | 13 | 16 |
| **Median** | 4 | 3 | 0 | 0 | 0 | 0 | 0 | 0 | 0 | 0 | 0 | 0 | 0 | 0 | 0 |
| Interquartile range | 4 | 5.75 | 1 | 0 | 0 | 0 | 0 | 1 | 2 | 1 | 0 | 0 | 0 | 0 | 0 |
| Lower quartile | 2 | 1 | 0 | 0 | 0 | 0 | 0 | 0 | 0 | 0 | 0 | 0 | 0 | 0 | 0 |
| Upper Quartile | 6 | 6.75 | 1 | 0 | 0 | 0 | 0 | 1 | 2 | 1 | 0 | 0 | 0 | 0 | 0 |

**Appendix V: Overall self-reported confidence score in performing procedures**

|  |  |  | **Sex** N=448 | |  |  |  | | **Type of medical School** N=449 | | | | | | |  | |  | | |
| --- | --- | --- | --- | --- | --- | --- | --- | --- | --- | --- | --- | --- | --- | --- | --- | --- | --- | --- | --- | --- |
| Procedure | **Overall Frequency** | **Percentage (%)** | **Female**  **n (%)** | **Male  n (%)** | **Fisher test X^2a^** | **p** | | **Public Federal medical schools** | | **Public State medical schools** | | **Private medical schools** | | **Fisher test X^2^*** | **p** | | **Confidence Ranking/15** | |  |  |
| **Urethral catheterization** |  |  |  |  |  |  |  | |  | |  | |  | | |  | |  |  |  |
| Confident^b^ | 253 | 56.5 | 83 (54.6) | 170 (57.4) | 0.327 | 0.568 | | 140 (53.2) | | 75 (63.6) | | 38 (55.9) | | 3.539 | 0.170 | | 1 | |  |  |
| Not confident | 195 | 43.5 | 69 (45.4) | 126 (42.6) |  |  | | 123 (46.8) | | 43 (36.4) | | 30 (44.1) | |  |  | |  | |  |  |
| **Intravenous Access** |  |  |  |  |  |  | |  | |  | |  | |  |  | | 2 | |  |  |
| Confident | 231 | 51.6 | 71 (46.7) | 160 (54.1) | 2.168 | 0.141 | | 123 (46.8) | | 68 (57.6) | | 40 (58.8) | | 5.591 | 0.061 | |  | |  |  |
| Not confident | 217 | 48.4 | 81 (53.3) | 136 (45.9) |  |  | | 140 (53.2) | | 50 (42.4) | | 28 (41.2) | |  |  | |  | |  |  |
| **Control of haemorrhage** |  |  |  |  |  |  | |  | |  | |  | |  |  | | 3 | |  |  |
| Confident | 141 | 31.5 | 46 (30.3) | 95 (32.1) | 0.156 | 0.693 | | 86 (32.7) | | 39 (33.1) | | 16 (23.5) | | 2.311 | 0.315 | |  | |  |  |
| Not confident | 307 | 68.5 | 106 (69.7) | 201 (67.9) |  |  | | 177 (67.3) | | 79 (66.9) | | 52 (76.5) | |  |  | |  | |  |  |
| **Vaginal delivery** |  |  |  |  |  |  | |  | |  | |  | |  |  | | 4 | |  |  |
| Confident | 116 | 25.8 | 38 (25.0) | 78 (26.4) | 0.096 | 0.757 | | 61 (23.2) | | 39 (33.1) | | 16 (23.5) | | 4.353 | 0.113 | |  | |  |  |
| Not confident | 332 | 74.2 | 114 (75.0) | 218 (73.6) |  |  | | 202 (76.8) | | 79 (66.9) | | 52 (76.5) | |  |  | |  | |  |  |
| **Suturing of lacerations*** |  |  |  |  |  |  | |  | |  | |  | |  |  | | 5 | |  |  |
| Confident | 100 | 22.3 | 23 (15.1) | 77 (26.0) | 6.859 | 0.009* | | 55 (20.9) | | 30 (25.4) | | 15 (22.1) | | 0.960 | 0.619 | |  | |  |  |
| Not confident | 348 | 77.7 | 129 (84.9) | 219 (74.0) |  |  | | 208 (79.1) | | 88 (74.6) | | 53 (79.1) | |  |  | |  | |  |  |
| **Wound Debridement** |  |  |  |  |  |  | |  | |  | |  | |  |  | | 6 | |  |  |
| Confident | 99 | 22.1 | 29 (19.1) | 70 (23.6) | 1.218 | 0.270 | | 52 (19.7) | | 26 (22.0) | | 21 (30.9) | | 3.881 | 0.144 | |  | |  |  |
| Not confident | 349 | 77.9 | 123 (80.9) | 226 (76.4) |  |  | | 211 (80.3) | | 92 (78.0) | | 47 (69.1) | |  |  | |  | |  |  |
| **Incision and drainage** |  |  |  |  |  |  | |  | |  | |  | |  |  | | 7 | |  |  |
| Confident | 74 | 16.5 | 18 (11.842) | 56 (18.919) | 3.647 | 0.056 | | 43 (16.4) | | 22 (18.6) | | 9 (13.2) | | 0.925 | 0.630 | |  | |  |  |
| Not confident | 374 | 83.5 | 134 (88.158) | 240 (81.081) |  |  | | 220 (83.7) | | 96 (81.4) | | 59 (86.8) | |  |  | |  | |  |  |
| **Uterine Evacuation** |  |  |  |  |  |  | |  | |  | |  | |  |  | | 8 | |  |  |
| Confident | 50 | 11.1 | 15 (9.9) | 35 (11.8) | 0.387 | 0.534 | | 32 (12.2) | | 11 (9.3) | | 7 (10.3) | | 0.724 | 0.696 | |  | |  |  |
| Not confident | 398 | 88.9 | 137 (90.1) | 261 (88.2) |  |  | | 231 (87.8) | | 107 (90.7) | | 61 (89.7) | |  |  | |  | |  |  |
| **Management of open fractures*** |  |  |  |  |  |  | |  | |  | |  | |  |  | | 9 | |  |  |
| Confident | 18 | 4.0 | 10 (6.6) | 8 (2.7) | 3.913 | 0.048* | | 9 (3.4) | | 4 (3.4) | | 5 (7.4) | | 2.329 | 0.312 | |  | |  |  |
| Not confident | 430 | 96.0 | 142 (93.4) | 288 (97.3) |  |  | | 254 (96.6) | | 114 (96.6) | | 63 (92.6) | |  |  | |  | |  |  |
| **Chest tube insertion** |  |  |  |  |  |  | |  | |  | |  | |  |  | | 10 | |  |  |
| Confident | 18 | 4.0 | 5 (3.3) | 13 (4.4) | 0.316 | 0.574 | | 9 (3.4) | | 5 (4.2) | | 4 (5.9) | | 0.872 | 0.647 | |  | |  |  |
| Not confident | 430 | 96.0 | 147 (96.7) | 283 (95.6) |  |  | | 254 (96.6) | | 113 (95.7) | | 64 (94.1) | |  |  | |  | |  |  |
| **Reduction of closed fractures** |  |  |  |  |  |  | |  | |  | |  | |  |  | | 11 | |  |  |
| Confident | 17 | 3.8 | 6 (3.9) | 11 (3.7) | 0.015 | 0.904 | | 11 (4.2) | | 2 (1.7) | | 4 (5.9) | | 2.350 | 0.309 | |  | |  |  |
| Not confident | 431 | 96.2 | 146 (96.1) | 285 (96.3) |  |  | | 252 (95.8) | | 116 (98.3) | | 64 (94.1) | |  |  | |  | |  |  |
| **Circumcision** |  |  |  |  |  |  | |  | |  | |  | |  |  | | 12 | |  |  |
| Confident | 14 | 3.1 | 5 (3.3) | 9 (3.0) | 0.021 | 0.886 | | 6 (2.3) | | 6 (5.1) | | 2 (2.9) | | 2.127 | 0.345 | |  | |  |  |
| Not confident | 434 | 96.9 | 147 (96.7) | 287 (97.0) |  |  | | 257 (97.7) | | 112 (94.9) | | 66 (97.1) | |  |  | |  | |  |  |
| **Caesarean section** |  |  |  |  |  |  | |  | |  | |  | |  |  | | 13 | |  |  |
| Confident | 13 | 2.9 | 5 (3.3) | 9 (3.0) | 0.119 | 0.731 | | 7 (2.7) | | 5 (4.2) | | 1 (1.5) | | 1.291 | 0.525 | |  | |  |  |
| Not confident | 434 | 97.1 | 147 (96.7) | 287 (97.0) |  |  | | 255 (97.3) | | 113 (95.8) | | 67 (98.5) | |  |  | |  | |  |  |
| **Hydrocelectomy** |  |  |  |  |  |  | |  | |  | |  | |  |  | | 14 | |  |  |
| Confident | 4 | 0.9 | 0 (0.0) | 4 (1.4) | 2.073 | 0.150 | | 2 (0.8) | | 2 (1.7) | | 0 (0.0) | | 1.526 | 0.466 | |  | |  |  |
| Not confident | 444 | 99.1 | 152 (100.0) | 292 (98.6) |  |  | | 261 (99.2) | | 116 (98.3) | | 68 (100.0) | |  |  | |  | |  |  |
| **Laparotom**y |  |  |  |  |  |  | |  | |  | |  | |  |  | | 15 | |  |  |
| Confident | 2 | 0.4 | 0 (0.0) | 2 (0.7) | 0.032 | 0.310 | | 2 (0.8) | | 0 (0.0) | | 0 (0.0) | | 1.421 | 0.491 | |  | |  |  |
| Not confident | 446 | 99.6 | 152 (100.0) | 294 (99.3) |  |  | | 261 (99.2) | | 118 (100.0) | | 68 (100.0) | |  |  | |  | |  |  |

^a^ Fishers exact test

^b^ "Confident" or "very confident" or “completely confident” was considered as confident.

*Statistically significant difference
